# Deep sequencing of Danish Holstein dairy cattle for variant detection and insight into potential loss-of-function variants in protein coding genes

**DOI:** 10.1186/s12864-015-2249-y

**Published:** 2015-12-09

**Authors:** Ashutosh Das, Frank Panitz, Vivi Raundahl Gregersen, Christian Bendixen, Lars-Erik Holm

**Affiliations:** Department of Molecular Biology and Genetics, Aarhus University, DK-8830 Tjele, Denmark

**Keywords:** NGS, Bovine, Genome, SNP, Indel, LoF

## Abstract

**Background:**

Over the last few years, continuous development of high-throughput sequencing platforms and sequence analysis tools has facilitated reliable identification and characterization of genetic variants in many cattle breeds. Deep sequencing of entire genomes within a cattle breed that has not been thoroughly investigated would be imagined to discover functional variants that are underlying phenotypic differences. Here, we sequenced to a high coverage the Danish Holstein cattle breed to detect and characterize single nucleotide polymorphisms (SNPs), insertion/deletions (Indels), and loss-of-function (LoF) variants in protein-coding genes in order to provide a comprehensive resource for subsequent detection of causal variants for recessive traits.

**Results:**

We sequenced four genetically unrelated Danish Holstein cows with a mean coverage of 27X using an Illumina Hiseq 2000. Multi-sample SNP calling identified 10,796,794 SNPs and 1,295,036 indels whereof 482,835 (4.5 %) SNPs and 231,359 (17.9 %) indels were novel. A comparison between sequencing-derived SNPs and genotyping from the BovineHD BeadChip revealed a concordance rate of 99.6–99.8 % for homozygous SNPs and 93.3–96.5 % for heterozygous SNPs. Annotation of the SNPs discovered 74,886 SNPs and 1937 indels affecting coding sequences with 2145 being LoF mutations. The frequency of LoF variants differed greatly across the genome, a hot spot with a strikingly high density was observed in a 6 Mb region on BTA18. LoF affected genes were enriched for functional categories related to olfactory reception and underrepresented for genes related to key cellular constituents and cellular and biological process regulation. Filtering using sequence derived genotype data for 288 Holstein animals from the 1000 bull genomes project removing variants containing homozygous individuals retained 345 of the LoF variants as putatively deleterious. A substantial number of the putative deleterious LoF variants had a minor allele frequency >0.05 in the 1000 bull genomes data set.

**Conclusions:**

Deep sequencing of Danish Holstein genomes enabled us to identify 12.1 million variants. An investigation into LoF variants discovered a set of variants predicted to disrupt protein-coding genes. This catalog of variants will be a resource for future studies to understand variation underlying important phenotypes, particularly recessively inherited lethal phenotypes.

**Electronic supplementary material:**

The online version of this article (doi:10.1186/s12864-015-2249-y) contains supplementary material, which is available to authorized users.

## Background

Identification of genetic variants underlying phenotypic traits is one of the major tasks associated with contemporary cattle genomic research. The traditional genome-wide association studies (GWAS) based on SNP genotyping technology is likely to have reduced power in detecting causal variants because of incomplete linkage disequilibrium with the genotyped SNPs [1]. This inefficiency has been reduced considerably by the advent of whole-genome sequencing technology. The technology has also been predicted to be an efficient approach to evaluate complex traits [2]. It is necessary to sequence at deep coverage to enable efficient and reliable detection of genetic variants using whole-genome sequencing. Sequencing at 15X coverage of the genome has been reported to enable identification of approx. 75 % of the genetic variants present in the heterozygote state [3]. An increase in sequence depth significantly improves both the accuracy and sensitivity of variant detection [4]. The accuracy of variant detection and detection rate is also dependent on the different variant detection algorithms [4].

Over the last few years, a number of bovine whole-genome sequencing studies have been carried out providing a substantial number of genetic variants in the form of single nucleotide polymorphism (SNP) and insertion/deletion (indel). These variants are catalogued in dbSNP [5] with input from the bovine HapMap project [6], the bovine genome project [7] and other whole genome sequencing studies on diverse cattle breeds [8–15]. Most recently, the 1000 bull genomes project has reported whole-genome sequencing of 234 bulls, leading to detection of 26.7 million SNPs and 1.6 million indels [16]. However, genome sequencing studies for mining genetic variants are still ongoing and a large fraction of genetic variants in different cattle breeds remains to be discovered and properly annotated.

Genetic variants detected using whole-genome sequencing has been used successfully to identify causal variants and to map complex traits in domestic cattle [16, 17]. In human, the causal role of loss-of-function (LoF) variants (defined as stop codon, splice site, frame-shift and large deletions in protein coding genes [18]) in severe Mendelian diseases is well established. Recently, Charlier et al. [17] showed that LoF variants at the homozygous state can compromise fertility in cattle by causing embryonic lethality [17]. Therefore, screening a comprehensive list of deleterious LoF variants detected by deep sequencing would be of considerable interest in cattle genomic studies targeting fertility and production traits.

In this study, we describe the results of whole-genome sequencing (at 27X coverage) of four unrelated (at least back to grandparents) Danish Holstein-Friesian cows. Multi-sample variant calling facilitated the detection of 12.1 million variants. The concordance of sequencing derived SNPs ranged from 93.3 to 99.8 % compared with the high-density chip data. We then generated a catalog of filtered LoF variants that will provide a resource for future functional studies on economically important traits thereby contributing to the contemporary cattle genomics research.

## Results and discussion

### Sequencing and mapping

Sequencing generated 3,035,569,908 reads of 100 bp for four Holstein cow genomes. Reads were mapped to the *Bos taurus* reference assembly UMD 3.1 [19] using the Burrows-Wheeler Aligner (BWA) [20]. The percentage of mapped reads ranged from 93.2 to 94.5 % with a mean of 94.1 % resulting in 288.5 Gb of data (Table 1). The mean depth of coverage was 27X (Table 1), which was in the same range as other deep re-sequencing studies [4, 8, 11, 12]. Per chromosome coverage by at least one sequencing read (Additional file 1) yielded average genome coverage of 98.7 % (Table 1). The higher percentage of genome coverage compared with previous studies [8, 10] could be explained by an increase in read lengths [21] as longer reads (100 bp) were utilized in this study.

**Table 1:** Summary of the alignment statistics for four Holstein cows genome

Animal ID	Number of total reads	Number of mapped reads	Number of bases in mapped reads	Mean depth	Genome coverage
44–6	766,207,530	720,413,573 (94.0 %)	72.8 Gb	27.3 X	98.7 %
46–25	731,266,080	691,737,221 (94.6 %)	69.9 Gb	26.2 X	98.7 %
49–25	766,453,890	724,694,871 (94.5 %)	73.2Gb	27.4 X	98.8 %
50–38	771,642,408	718,944,575 (93.2 %)	72.6 Gb	27.2 X	98.7 %
Total	3,035,569,908	2,855,790,240 (94.1 %)	288.5 Gb	27.0 X	

### SNP and indel detection

**Fig. 1:**
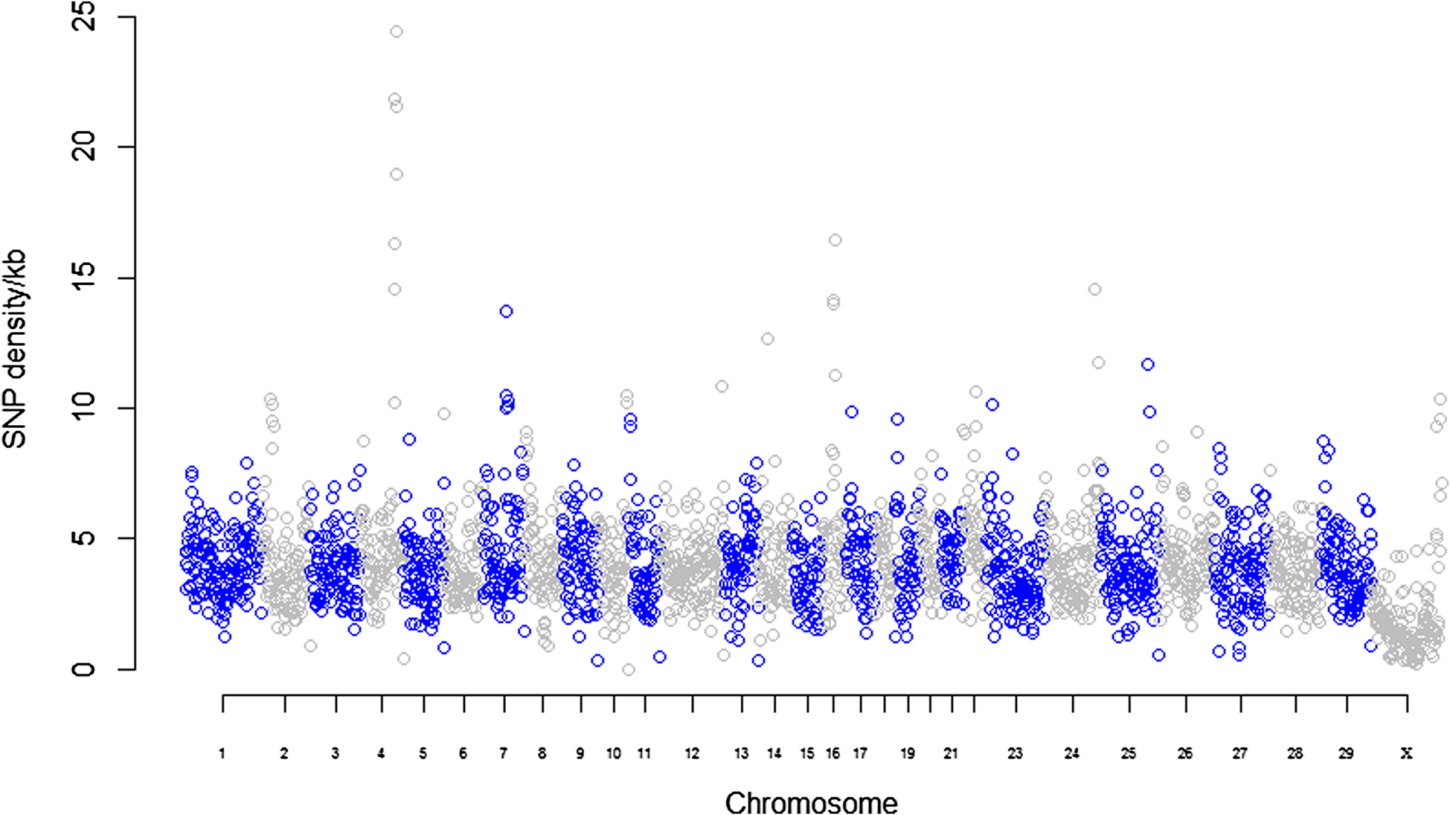
Genome-wide SNP densities. The plot was generated using SNP density per kb on y-axis for a bin size of 1 Mb on each chromosome (x-axis; chromosome indicated)

Multi-sample variant calling using the UnifiedGenotyper from the Genome Analysis Toolkit (GATK) [22] identified 10,796,794 SNPs of which 53 % were heterozygous. 482,835 SNPs (4.5 %) were novel and 10,313,959 SNPs (95.5 %) were known compared with dbSNP (build 140) (Table 2). Most of the SNPs (99.9 %) were biallelic while the remaining 0.1 % was triallelic. The genome-wide mean transition to transversion ratio was 2.11, which is in agreement with previous Holstein genome re-sequencing studies [14]. The SNP density across the genome (Fig. 1) showed a uniform chromosomal distribution of SNPs in line with the findings by Kawahara-Miki et al. [8]. Multi-sample indel calling using UnifiedGenotyper from GATK [22] resulted in the identification of 1,295,036 indels (−58 to +66 bp) of which 231,359 (17.9 %) were novel while the remaining are previously described in dbSNP (build 140). 50.7 % of the indels represent deletions while the remaining 639,094 (49.3 %) were insertions (Table 2). In order to reduce the risk of eliminating true genetic variants present at a low frequency, we used a less stringent filtering approach resulting in the number of detected SNPs per animal (Additional file 2) being higher than detected in previous studies on Holstein cattle [4, 13, 14]. The use of different variant detection algorithms could also influence the number of SNPs discovered [4]. However, the number of detected SNPs within an individual was lower in this study than in a previous study in the Old Danish Jutland breed using the same algorithm and filtering parameters [23]. This lower level of genetic variation could be an effect of the long period of artificial selection with a low effective population size in the Danish Holstein breed [24].

**Table 2:** Summary statistics of the identified variants

Total SNPs	10,796,794
Homozygous SNPs	5,078,645 (47.0)
Heterozygous SNPs	5,718,149 (53.0)
Novel SNPs	482,835 (4.5)
Known SNPs	10,313,959 (95.5 )
Biallelic SNPs	10,780,608 (99.9)
Triallelic SNPs	16,186 (0.1)
Ts:Tv	2.11:1.00
Total indels	1,295,036
Heterozygous indels	566,749 (43.8)
Homozygous indels	728,287 (56.2)
Novel indels	231,359 (17.9)
Known indels	1,063,677 (82.1)
Deletions	655,942 (50.7)
Insertions	639,094 (49.3)

Values in parentheses are the percentage of variants in the specific class of the total variants that type of variant

### Evaluation of variant calling

Samples used for sequencing were also genotyped using the BovineHD BeadChip (Illumina Inc., San Diego, CA) to evaluate the accuracy of SNP detection and genotype calling from sequencing data. Mitochondrial SNPs and SNPs with ambiguous chromosomal positions on the UMD 3.1 assembly [19] were filtered from the array calls. SNP genotypes retained on 29 autosomes and chromosome X (Additional file 3) were compared to the sequencing derived SNPs. 99.7–99.9 % of the homozygous alternative SNP calls in the BovineHD BeadChip were identified in our sequencing calls. The rate of genotype concordance was 99.6–99.8 % (Table 3). The detection rate was 93.7–96.7 % for the heterozygous SNPs and the rate of concordant calls was 93.3–96.5 % (Table 4). The discordant SNPs were classified into different categories: 1) Homozygous SNPs over-called as heterozygous due to sequence errors in the reads or erroneously mapped reads; 2) heterozygous SNPs under-called as homozygous due to insufficient sequencing depth; 3) SNPs called as homozygous both on the BovineHD BeadChip and in the sequencing call but with different alleles were designated as inconsistent calls and could be a result of ambiguous mapping of reads or sequence errors in the reads. We observed a very low rate at which homozygous SNPs over-called as heterozygous (0.1–0.2 %), heterozygous SNPs under-called as homozygous (0.2–0.4 %) and of inconsistent calls (≤4 per cow) (Tables 3 and 4). A comparison with the results of a previous report by Zhan et al. [4] revealed that we had a lower rate of inconsistent calls in our results maybe as a consequence of higher sequencing depth and use of a different SNP calling algorithm.

**Table 3:** Comparison of BovineHD chip homozygous alternative genotypes to sequencing calls

Animal ID	BovineHD	Sequencing calls	Concordant	Inconsistent	Homozygous > heterozygous
44–6	247,562	247,519 (99.9 %)	247,237 (99.8 %)	17	265 (0.1 %)
46–25	255,886	255,296 (99.8 %)	254,826 (99.6 %)	3	467 (0.2 %)
49–25	259,773	259,240 (99.8 %)	259,001 (99.7 %)	4	235 (0.1 %)
50–38	255,427	254,729 (99.7 %)	254,407 (99.6 %)	4	318 (0.1 %)

Concordant, the same alleles at the same sites were detected by both the BovineHD chip and sequencing calls; Inconsistent, homozygous calls by both the Bovine HD chip and sequencing calls but with different alleles; Homozygous > heterozygous, homozygous SNPs on the BovineHD chip that were over-called as heterozygous in sequencing calls

**Table 4:** Comparison of BovineHD chip heterozygous genotypes to sequencing calls

Animal ID	BovineHD	Sequencing calls	Concordant	Heterozygous > homozygous
44–6	223,103	215,822 (96.7 %)	215,348 (96.5 %)	474 (0.2 %)
46–25	225,985	214,607 (94.9 %)	214,065 (94.7 %)	542 (0.2 %)
49–25	215,929	204,225 (95.6 %)	203,639 (94.3 %)	586 (0.3 %)
50–38	222,315	208,328 (93.7 %)	207,498 (93.3 %)	830 (0.4 %)

Concordant, the same alleles at the same sites were detected by both the BovineHD chip and sequencing calls; Heterozygous > homozygous, heterozygous SNPs on the BovineHD chip that were under-called as homozygous in sequencing calls

To validate the indel calls, PCR primers and probes were designed for 10 indels. PCR products were used to genotype 90 Danish Holstein breeding bulls. Genotyping was performed by size determination on a 3730XL DNA analyzer and the data was analyzed using Genemapper v.3.7 (Applied Biosystems, Foster City CA, USA). Of the 10 indels genotyped, 9 indels were found to be true indels.

### Functional annotation of variants

**Fig. 2:**
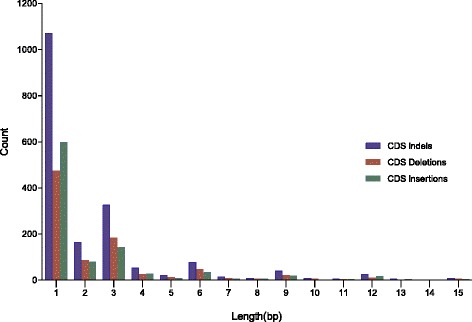
Characteristics and length distribution of Indels (≤15 bp) in coding sequence (CDS). The horizontal axis shows the length of indels and the vertical axis indicates the count of indels

Annotation using NGS-SNP [25] assigned a range of functional classes to the variants identified (Table 5). Most of the variants were located in intergenic regions. Intronic variants represent the majority of the variants located in genic regions whereas the regions 5 kb upstream and downstream of a transcript exhibited a total of 772,334 variants. 27,644 variants were located in the 5′ or 3′ untranslated regions. The number of variants identified in splice sites included 283 splice donor and 300 splice acceptor site variants. In addition, 7289 variants causing a change within the region of the splice site (1–3 bases into an exon or 3–8 bases into an intron) were found. In total, 74,886 SNPs were predicted to affect the coding sequences: 395 SNPs were predicted to cause a premature stop codon, 29 to destroy a termination codon, and 34,257 (45.74 %) to cause non-synonymous substitutions (34,183 missense and 74 SNPs predicted to change at least one base of the first codon of a transcript). The remaining 40,180 SNPs in the coding sequences were predicted to have either synonymous or indeterminate effects. In total 1937 indels were identified in coding sequences: 1302 indels predicted to cause a disruption of the translational reading frame, 194 indels predicted to cause inframe insertions, 261 to cause inframe deletions, 44 to create amino acid changes in the encoded protein without changing the frame, one to destroy the initiation codon and 137 to have an indeterminate effect. We observed that indels in coding sequences were enriched for lengths that are a multiple of three (3n) (Fig. 2) suggesting a purifying selection against frame-shift in coding sequences as has been observed by Daetwyler et al. [16]. Considering SNPs and indels jointly 3984 variants were identified in non-coding genes: 3839 in non-coding exons, 99 in mature miRNAs and 46 in non-coding RNAs. Annotation revealed that the proportion of non-synonymous nucleotide substitutions, splice site and frameshift variants was lower in Danish Holstein than in the Japanese Kuchinoshima-Ushi [8] and Korean Hanwoo breeds [10] in agreement with the previous studies on these two breeds.

**Table 5:** Annotation of variants by functional class

Functional class	SNP	Indel
Intergenic	7,345,721 (68.0)	866,042 (66.9)
Intronic	2,656,868 (24.6)	334,542 (25.8)
Upstream	367,709 (3.4)	46,226 (3.6)
Downstream	317,069 (2.9)	41,330 (3.2)
3′ UTR	19,677 (0.2)	3150 (0.2)
5′ UTR	4364 (0.0)	453 (0.0)
Splice region^a^	6393 (0.1)	896 (0.1)
Splice donor^b^	230 (0.0)	53 (0.0)
Splice acceptor^c^	218 (0.0)	82 (0.0)
Initiator codon^d^	74 (0.0)	1 (0.0)
Stop gain	395 (0.0)	-
Frameshift	-	1302 (0.1)
Missense	34,183 (0.3)	44 (0.0)
Synonymous	40,055 (0.4)	-
Coding sequence^e^	125 (0.0)	135 (0.0)
Inframe deletion	-	261 (0.0)
Inframe insertion	-	194 (0.0)
Stop lost	29 (0.0)	-
Stop retained	25 (0.0)	-
Within non coding exon^f^	3569 (0.0)	270 (0.0)
Within mature miRNA	70 (0.0)	29 (0.0)
Nc transcript^f^	20 (0.0)	26 (0.0)
Total	10,796,794 (100.0)	1,295,036 (100.0)

^a^Variant in which a change has occurred within the region of the splice site either within 1–3 bases of the exon or 3–8 bases of the intron

^b^Variant is located in the first two bases of an intron

^c^SNP is located in the last two bases of an intron

^d^SNP changes at least one base of the first codon of a transcript

^e^SNP is located in coding sequence with indeterminate effect

^f^SNP is a transcript variant of a non-coding RNA. Values in parentheses are the percentage of variants in the functional class of the total variants in the column

### LoF variants in protein-coding genes

**Fig. 3:**
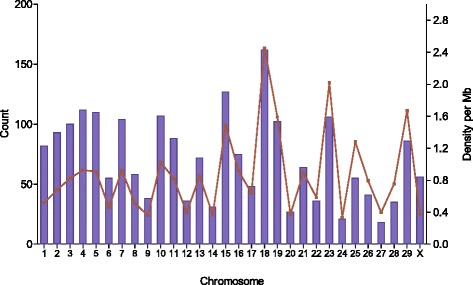
Distribution and densities of LoF variants across the genome. The blue bars on the X axis represent the number of putative deleterious LoF variants in each chromosome whereas the red line indicates the densities of LoF per Mb on the chromosome

We identified 2145 LoF variants in 1453 protein-coding genes in total including 395 stop gains in 345 genes, 448 splice site variants (splice donor and splice acceptor sites) in 392 genes and 1302 frame-shift indel variants in 931 genes (Table 6). There were 215 genes harboring more than one LoF variant. Of these 2145 LoF variants, 1431 were heterozygous at least in one of the sequenced animals while the remaining 714 variants were homozygous in all four sequenced animals. These homozygous variants in all four animals might be breed specific variation, or it might be due to errors in the reference sequence. In total 1568 (73 %) of the LoF variants were not present in dbSNP build 133. Investigation on the distribution and densities of these LoF variants show the highest density on BTA 18 (Fig. 3), particularly in genes spanning the 57–64 Mb region including aldehyde dehydrogenase family 16 member A1 (*ALDH16A1*) and uncharacterized proteins (ENSBTAG00000006859, ENSBTAG00000037699, ENSBTAG00000011844, ENSBTAG00000040392, ENSBTAG00000000336, ENSBTAG00000014953, ENSBTAG00000009171). A comparison with annotated variants in the Danish Jutland breed [23] revealed a similar rate of LoF variants in this region. However, tandem duplications (TD) and inversions (INV) identified in this region (Additional file 4) suggest that the higher densities of LoF might be influenced by structural variants.

**Table 6:** Numbers of LoF variants before filtering and putative deleterious LoF variants after filtering

Variant type	Before filtering				After filtering		
	Total (novel)	Gene count	LoF^hom^	LoF^het^	AH^1^	Consistent	Inconsistent
Stop gain	395 (210)	345	28	367	97	97	0
Splice site	448 (235)	392	72	376	77	76	1
Frameshift indel	1302 (1123)	931	614	688	171	95	76
Total	2145 (1568)		714	1431	345	268	77

LoF^hom^ variants were homozygous in all four sequenced Danish Holstein cows; LoF^het^ variants for which at least one of the four sequenced cows was heterozygous; AH^1^ LoF variants for which none of the 288 sequenced Holstein animals from the 1000 bull genomes project was homozygous; Consistent are concordantly called both in the four Danish Holstein cows and the 288 Holstein animals; Inconsistent variants called as discordant variant types (SNP as indel or indel as SNP) between the four Danish Holstein cows and the 288 bulls from the 1000 bull genomes project; Novel variants are not annotated in dbSNP build133

Gene ontology (GO) enrichment analysis using the PANTHER version 10.0 [26] revealed that genes related to “olfactory receptor activity”, “detection of chemical stimulus involved in sensory perception of smell”, “sensory perception of smell”, “detection of chemical stimulus involved in sensory perception”, “detection of stimulus involved in sensory perception” and “detection of chemical stimulus” were overrepresented in LoF affected genes (Additional file 5). Overrepresentation of these GO terms could be explained by the enrichment of olfactory receptor genes in the group of LoF affected genes. Olfactory receptor genes form the largest multigene family in mammals (with more than 2000 copies of intact genes, pseudogenes and truncated olfactory receptor genes in the bovine genome) [27] making it probable that the occurrence of numerous copies of specific domains creates problems in mapping of short sequences resulting in erroneous identification of genetic variation within these related genes. The GO terms “G-protein coupled receptor activity”, “transmembrane signaling receptor activity”, “neurological system process”, “signaling receptor activity”, and “G-protein coupled receptor signaling pathway” were also overrepresented in LoF affected genes. G-protein coupled receptors (GPCRs) are known to be associated with neurotransmission, transmembrane receptor activity and signaling pathways [28]. GPCRs are the largest family of membrane proteins encompassing ~2 % of the human proteins [29]. At present, the number of GPCR protein domains listed in the Ensembl database for human and cow are 53 and 52, respectively. The loss of a GPCR might be deleterious to a particular function in which it is involved, however it has no apparent effect in other cases [30]. These observations support a LoF tolerance for specific members of GPCRs explaining the overrepresentation of GPCRs within the group of LoF affected genes in this study. A similar enrichment for LoF containing genes implicating olfactory reception and GPCR receptor activity were also observed in the human data published by MacArthur et al. [18]. In contrast, genes related to GO terms “membrane-bounded organelle”, “cytoplasm”, “intracellular part”, “organelle”, “intracellular membrane-bounded organelle”, “intracellular organelle”, “ cytoplasmic part” and “ cell ” were significantly (*P* < 0.05) underrepresented in the set of LoF affected genes (Additional file 6). These GO terms cover almost all important constituent parts of a cell [31]. Eukaryotic nuclear organization plays important roles in the coordination of transcription with subsequent processes involved in gene expression [32], whereas ribosomal biogenesis provide the framework in the eukaryotic translation mechanism [33]. Proper cooperation between the plasma membrane, the nucleus and other organelles such as mitochondria and the endoplasmic reticulum is crucial for many cellular processes including synthesis and intracellular transport, intracellular homeostasis, controlling fundamental processes like motility and contraction, secretion, cell growth, proliferation and apoptosis [34]. Moreover, mutational disruption of genes related to nuclear protein [35], ribosomal biogenesis [36], mitochondrial DNA [37], endoplasmic reticulum [38], golgi apparatus [39] and plasma membrane [40] are associated with recessively inherited disorders in mammals. Taken together these observations support a possible selection against the occurrence of LoF variants in genes related to the nucleus, ribosomes, mitochondria, the endoplasmic reticulum, the golgi apparatus and the plasma membrane. LoF affected genes were also depleted for the GO terms “positive regulation of cellular process”, “cellular metabolic process”, “protein binding” and “positive regulation of biological process”. Previous studies have shown that loss of the function of genes related to these annotations are associated with inherited diseases in mammals [41–44]. These observations make it probable that selection against LoF mutations within these genes explains the reduced occurrence of LoF within these genes in our study. A significant depletion for genes related to “protein binding”, “intracellular membrane-bounded organelle”, cytoplasmic part”, “intracellular organelle part” and “nuclear part” was also reported by MacArthur et al. [18]. There were overlaps for LoF containing genes such as olfactory receptor, family 52, subfamily N, member 4 (*OR52N4*) and 1-acylglycerol-3-phosphate O-acyltransferase 2 (*AGPAT*2) between our data and the data from MacArthur et al. [18]. The two data sets also included closely related gene family members (paralogs) for instance, ATP-binding cassette, sub-family A (ABC1), member 12 and 13 (cattle *ABCA12* vs human *ABCA13*); kallikrein-related peptidase 9 and 12 (cattle *KLK9* vs human *KLK12*); tigger transposable element derived 6 and 7 (cattle *TIGD6* vs human *TIGD7*); uridine diphosphoglucuronosyltransferase 2, poly peptide B15 and 17 (cattle *UGT2B15* vs human *UGT2B17*); and zinc finger protein 648 and 671 (cattle *ZNF648* vs human *ZNF681*). These observations give an indication that a similar LoF tolerance exists for members of these gene families implicating the potential for rescuing LoF mutations by other genes within the gene family both in cattle and human.

Recent studies reported an increased rate of false-positive calls among LoF variants [18, 45]; therefore it is necessary to test the accuracy of their identification. To test the false-positive rate of our LoF variants, 34 SNPs (15 stop gain and 19 splice site variants) and 24 frame-shift indels were selected for genotyping. A group of 90 Danish Holstein breeding bulls in addition to the four sequenced cows was genotyped for the selected variants. SNP genotyping using custom Taqman SNP genotyping assays and indels genotyped by size determination of PCR products revealed four (7.1 %) variants were false positive, while two assays did not produce PCR products (Additional file 7). This finding suggests that the LoF variants list will require more filtering and experimental validation (like Sanger sequencing of variable animals) to generate a high-confidence data set.

Heterozygous LoF variants segregating in the absence of homozygotes in a population could be imagined to encompass deleterious variants including causal variants for embryonic/fetal death. We got access to the data on genotypic variation from whole-genome resequencing data of 288 Holstein animals from the 1000 bull genomes project after completion of run four of this project [4]. These animals are expected to represent a major fraction of the genetic diversity in the global Holstein population. Accessibility to such a large data set allowed us to perform a filtering based on the absence of homozygotes for the variant allele of the identified LoF variants. Filtering retained 345 variants (97 stop-gain, 77 splice-site and 171 frame-shift indels) as putative deleterious from our list of LoF variants (Table 6). Information on these 345 putative deleterious LoF variants is presented in Additional file 8. Enrichment of GO terms related to olfactory reception remains significant for the genes in the list of putative deleterious LoF variants (Additional file 9). However, GO terms related to GPCRs were not significantly enriched in this list in contrast to the complete list of 2145 LoF variants suggesting that LoF mutations in GPCRs either are tolerated or can be rescued by gene products from other genes within this gene family. GO terms related to key cellular constituents and cellular and biological process regulation remain significantly depleted for genes affected by putative deleterious variants emphasizing that genes associated with these GO terms are less likely to tolerate LoF mutations.

We then tested the 345 filtered LoF variants for concordance of the class of genetic variants called in both the 1000 bull genome and our data set and found that 268 variants were concordantly called. However, 77 positions had an inconsistent call between the two data sets; particularly 76 were called as indels in our data but were called as a SNP in the 1000 bulls data (Table 6). These inconsistent calls might be attributable to differences in the variant detection algorithm [4] as we used UnifiedGenotyper from GATK [22] whereas the 1000 bull genomes project used mpileup from SAMtools [46]. Of the 76 (SNP vs indel) inconsistent calls, 64 were annotated as indels in dbSNP build 140, but as these indels might have been detected using the same algorithm as used in our study this is not necessarily corroborating our interpretation. We, therefore, tested 15 inconsistent calls by sequencing PCR products (Additional file 10) to validate the nature of the genetic variant. Of the 15 tested variants, 11 were found to be indels whereas none was confirmed as a SNP. Four inconsistent calls were confirmed as false positive that could be a result of ambiguous mapping of reads. Taken together these results suggest that GATK is providing more accurate calls than Samtools, which is consistent with observations from previous studies [47, 48]. UnifiedGenotyper from GATK has also been found to be an accurate variant caller in identification and validation of LoF variants in clinical contexts [49].

**Fig. 4:**
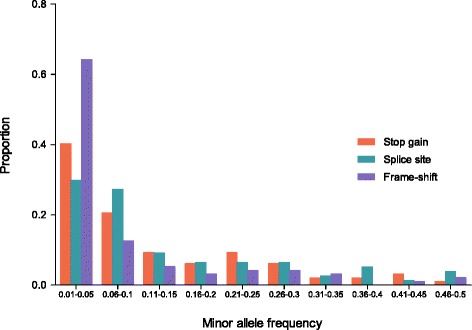
Minor allele frequency distribution for putative deleterious LoF variants called by both GATK and SAMtools. Minor allele frequency was calculated using data for the 288 Holstein animals from the 1000 bull genomes project

The minor allele frequency (MAF) distribution of the 268 concordantly called LoF variants (Fig. 4) using the 1000 bull genome data set show that MAF is >0.05 for more than 60 % of the stop-gain SNPs and splice site variants while less than 40 % of the frame-shift indels show a MAF >0.05. It was recently demonstrated that balancing selection allows a variant that is lethal in homozygotes to persist in the population at a relatively high frequency [50], thus variants with minor allele frequency >0.05 could be of considerable interest for further studies to mine causative mutations for recessive traits/disorders.

The list of putative deleterious LoF variants can be used to mine candidate regions from association studies for potential deleterious candidate variants. However, the candidates from the list should be validated experimentally by Sanger resequencing to confirm the actual presence of the variant and the gene models used for predicting the annotation should be investigated to confirm the annotation. Likewise, gene expression studies should be conducted to validate the consequences of the splice site variants. The list of putative deleterious LoF variants will despite the necessity of experimental validation be valuable information for future studies on detecting causal variants for recessive lethal or deleterious phenotypes in Holstein cattle also because of the similarity with results in human [18].

## Conclusions

The re-sequencing of four Danish Holstein cow genomes with a mean depth of 27X coverage followed by multi-sample variant calling identified a total of 12.1 million variants. The SNP detection rate and genotype concordance compared with high density chip data indicate high-confidence of the SNP calls. Functional annotation of identified SNPs showed that the proportion of non-synonymous substitutions was lower than those identified in genetically distant cattle breeds. A comparison of the results obtained in this study with the results from our previous study [23] using the same variant detection algorithm and filtering parameters revealed a reduced number of genetic variants segregating in Danish Holsteins compared to the Old Danish Jutland cattle. The reduction in genetic variation is probably a consequence of the increased inbreeding level caused by the high selection imposed on Danish Holstein [24].

An investigation into LoF variants revealed the highest density of putative deleterious LoF variants on BTA18, particularly in genes within the region spanning 57–64 Mb. This higher density might be associated with structural variants segregating in this region. Genes affected by putative deleterious LoF were strongly enriched for functional categories related to olfactory reception and depleted for genes related to key cellular constituents, cellular and biological process regulation. A comparison between our data and data from MacArthur et al. [18] provided an indication that a similar LoF tolerance pattern exists for genes both in cattle and human. Filtering using data for 288 animals from the 1000 bull genomes project revealed 345 LoF variants where none of the sequenced animals was homozygous. 268 of them were concordantly called between the two data sets (the four Danish Holstein vs. the 1000 bulls genome data). We observed 77 inconsistent calls (indel vs SNP) between our filtered data set and the 1000 bull genomes data, which could be a consequence of the use of different variant calling algorithms (GATK vs SAMtools). Sequencing PCR products for 15 inconsistent calls revealed that GATK provided more accurate calls than SAMtools. More than 60 % of the concordantly called LoF SNPs while less than 40 % of the frame-shift indels were observed to have a minor allele frequency >0.05 in the 1000 bull genomes data set. In future studies, it will be worthwhile to examine whether any one of these identified LoF variants is compromising fertility by recessive embryonic/prenatal lethality in the global Holstein population.

## Methods

### Library preparation and sequencing

We selected four unrelated (at least back to grandparents) Danish Holstein cows representing the genetic diversity of the Danish Holstein-Friesian breed based on pedigree records from Danish breeding animals. Genomic DNA was extracted from ear tissue of selected cows using a modified salting out method [51]. Paired-end libraries with different insert sizes (300 and 800 bp) were prepared using the protocol provided by Illumina. Sequencing of DNA was performed using an Illumina Hiseq 2000 (Illumina Inc., San Diego, CA).

### Animal ethics

All procedures were approved by the National Guidelines for Animal Experimentation and the Danish Animal Experimental Ethics Committee, and all sampling was restricted to routine on-farm procedures that did not cause any inconvenience or stress to the animals and hence no specific permissions was required.

### Processing of sequenced data

The quality of the sequence data was assessed using FastQC version 0.10.0 (http://www.bioinformatics.babraham.ac.uk/projects/fastqc/). The primary processing and filtering of the sequence data was performed using the FASTX-Toolkit version 0.0.13 [52] using the option ‘–Q33” in FASTX-Toolkit to convert the Illumina quality to Sanger quality. After quality filtering, the sequence reads of each animal was separately mapped to the *Bos taurus* genome assembly UMD 3.1 [19] using BWA version 0.5.9 [20] with default parameters. All output SAM files were converted to BAM files using SAMtools version 0.1.18 [46]. BAM files for all animals were merged into a sorted single BAM file using Picard version 1.86 (http://broadinstitute.github.io/picard/) with options “USE_THREADING = true, VALIDATION_STRINGENCY = LENIENT, SO = coordinate, ASSUME_SORTED = true, and CREATE_INDEX = true”. The Picard command CollectMultipleMetrics was employed to each of the merged bam files to provide information on alignment summary metrics, insert size metrics and quality by cycle metrics. Duplicates in BAM files were marked by applying MarkDuplicates from Picard. Genome coverage of each merged BAM file was estimated using BEDtools version 2.15.0 [53].

### Variant calling and filtering

RealignerTargetCreator and IndelRealigner from the GATK version 2.4.7 [54] were applied for local realignment around known indels from dbSNP build 133. Realigned BAM files were subjected to base quality recalibration using BaseRecalibrator from the GATK and the recalibration report for each realigned BAM file was generated using default setting for covariates and dbSNP build 133 as a variant database for known sites. Finally, recalibrated BAM files were generated using PrintReads from the GATK. Multi-sample SNP calling were performed using UnifiedGenotyper [22] from the GATK with options “--min_base_quality_score 20”, “-stand_call_conf 30”, “-stand_emit_conf 30”, “-dcov 200” and default settings for other parameters. Bovine genetic variants (8,757,145 SNPs and 519,609 indels) from dbSNP build 133 [5] were incorporated in SNP calling to populate the ID column of the known SNPs. Filtering of generated variants was performed using VCFtools version 0.1.8 [55] to remove variants mapped on unplaced scaffolds of the genome keeping only those mapped to chromosomes. The transition: transversion ratio and SNP density across the genome were obtained with 1 Mb bin size using VCFtools [55]. Multi-sample indel calling was performed separately using UnifiedGenotyper from the GATK [22] with options “--genotype_likelihoods_model INDEL”, “--min_indel_count_for_genotyping 5” and “--min_indel_fraction_per_sample 0.25”. Other parameter settings were similar to the SNP detection described above.

### Validation of sequencing-derived SNPs

To validate the sequencing-derived SNPs, the same four cows were genotyped using the BovineHD BeadChip (Illumina Inc., San Diego, CA) containing 777,962 SNPs. Chromosomal positions of the SNPs was determined according to the *Bos taurus* genome assembly UMD 3.1 [19]. Mitochondrial SNPs and SNPs with ambiguous chromosomal positions were filtered out from the array calls. Retained SNP genotypes (on 29 autosomes and chromosome X) from the BovineHD array were used in the subsequent calculation of the SNP detection rate. The detection rate for homozygous SNPs was calculated as the percentage of SNPs successfully genotyped as homozygous alternative on the BovineHD array that were concordantly called in sequencing (because homozygous alleles identical to reference sequence were not listed by sequencing-derived calls). The detection rate of heterozygous SNPs was calculated in a similar manner, as the percentage heterozygous calls in BovineHD array data that were concordantly called in sequencing. The discordant SNPs were separated in different categories based on their discrepancy.

### Validation of sequencing-derived indels

To validate novel functional indels, we designed PCR primers for 10 indels. Primers are listed in Additional file 11. Genotyping was performed by size determination of PCR products using 3730XL DNA analyzer and data were inspected using Genemapper software v.3.7 (Applied Biosystems, Foster City CA, USA).

### Annotation of variants

We used NGS-SNP [25] for functional annotation of identified SNPs and indels. The details of the resulting annotation fields have been described by Grant et al. [25]. Briefly, NGS-SNP provides rich annotations for genome-wide SNP and indels in organisms for which reference sequences are available in the Ensembl database. It reports a “Model_Annotations” field with detailed comparisons of SNP/indel to an orthologous gene typically in a well-characterized species. NGS-SNP also classifies whether or not the amino acid change is deleterious based on SIFT [56] prediction. Other important fields include overlapping protein features or domains, gene ontology information, and the conservation of both the SNP site and flanking sequence compared to a well-characterized species. NGS-SNP also reports NCBI, Ensembl, and UniProt IDs for genes, transcripts, and proteins when applicable. A gene description, phenotypes linked to the gene and whether the SNP/indel is novel or known is also supplemented in the annotated field. In our analysis, NGS-SNP utilized information from Ensembl release 72 [57], dbSNP Build 133 [58], Entrez Gene [58] and UniProt release 2013_09 [59]. We incorporated *Homo sapiens* as the model species for sequence conservation during annotation because most of the eukaryotic genes are well characterized in human.

### Gene ontology (GO) enrichment analysis

The list of genes predicted to be affected by putative deleterious LoF variants was subjected to gene ontology enrichment analysis using the PANTHER Overrepresentation Test (release 20150430) from the PANTHER version 10.0 [26]. All genes (*Bos taurus*) in the PANTHER database was used as the reference list in our analysis. The analysis set comprised the Ensembl IDs for 1453 genes of which 1308 were present in the reference list while the remaining 108 genes were not found. Therefore, our enrichment analysis represents results for 1308 genes predicted to be affected by LoF variants. Annotation data sets used were the complete GO biological process, GO cellular component and GO molecular function. In our analysis, we used default settings with Bonferroni correction for multiple testing. GO analysis for 345 putative deleterious variants was performed in a similar setup using the corresponding gene list for 345 variants.

### Genotyping of LoF variants

Custom Taqman SNP genotyping assay (Applied Biosystems, Foster City, CA, USA) was used to test the false positive rate (FPR) of novel functional SNPs. We used a subset of 50 novel SNPs identified by sequencing for genotyping using DNA samples from 90 Danish Holstein breeding bulls. Genotyping was performed on a ViiA7 Real-Time PCR System (Applied Biosystems, Foster City CA, USA). To test FPR for frame-shift indels, 90 bulls were genotyped for 24 indels using size determination of PCR products. Primers and probes are listed in Additional file 7.

### Testing inconsistent calls

To validate the nature of the genetic variant 15 inconsistent calls between our data and the 1000 bull genomes project data were tested by sequencing of PCR products using a 3730XL DNA analyzer (Applied Biosystems, Foster City CA, USA) and data were analyzed using the CodonCode^TM^ Aligner-Software (LI-COR, Inc., Lincoln, USA) version 3.7.1. Primers are listed in Additional file 10.

## Availability of supporting data

Whole-genome sequencing data from the four cows have been deposited at NCBI’s Short Read Archive (SRA) under the accession numbers SRX1370285-6, SRX1369736, and SRX1364945. The identified SNPs have been deposited at the Database of Short Genetic Variations (dbSNP) with accession numbers ss1947222024-1958018817 and indels with accession numbers ss1958018838-1960972587. Genotype data used in this study are from the 1000 Bull Genome Project [16].

## Additional files

Additional file 1:
**Per chromosome coverage by sequencing reads.** The horizontal axis shows 29 autosomes and X chromosome of the reference genome while the left vertical axis indicates scale of the chromosome length in Mbp. (PDF 23 kb) Additional file 2:
**Numbers of genetic variants identified per animal.** (XLSX 9 kb) Additional file 3:
**Summary of SNP genotyping per animal using the BovineHD BeadChip.** (XLSX 9 kb) Additional file 4:
**Structural variants in BTA 18 (TD tandem duplications and INV inversions).** Vertical grey bars indicate heterozygous variants while the red bars indicate homozygous variants. The blue mark highlighted region represents BTA18:57–64 Mb. (PDF 8 kb) Additional file 5:
**Gene ontology terms significantly (** ***p*** **< 0.05) overrepresented among genes affected by LoF variants.** p values were gene rated by Bonferroni correction for multiple tests. (XLSX 13 kb) Additional file 6:
**Gene ontology terms significantly (** ***P*** **< 0.05) underrepresented among genes affected by LoF variants.** p values were generated by Bonferroni correction for multiple tests. (XLSX 19 kb) Additional file 7:
**List of LoF variants used in validating genotyping.** This table also presents the list of primers used in the custom SNP genotyping assay and primers used in genotyping of frameshift indels by size determination. For frameshift indels forward primers were 5′ modified with reporter dyes (dyes are indicated in the parenthesis). (XLSX 21 kb) Additional file 8:
**List of 345 putative deleterious LoF variants.** (XLSX 31 kb) Additional file 9:
**Gene ontology terms significantly enriched or depleted in 345 putative deleterious LoF variants affected genes.** p values were generated by Bonferroni correction for multiple tests. (XLSX 13 kb) Additional file 10:
**List of inconsistent calls and corresponding primers used in validating by sequencing.** (XLSX 11 kb) Additional file 11:
**List of the primers and probes used to validate sequencing derived indels.** (XLSX 9 kb)

## Abbreviations

BTA: *Bos taurus* autosome
FPR: False positive rate
GATK: Genome analysis tool kit
Gb: Gigabase
GO: Gene ontology
GWAS: Genome-wide association study
INV: Inversion
kb: Kilobase
LoF: Loss-of-function
miRNA: Micro RNA
NCBI: National Center for Biotechnology Information
PCR: Polymerase chain reaction
SNP: Single-nucleotide polymorphism
TD: Tandem duplication
UTR: Untranslated region
