# Supplementary figures and images for: Deep sequencing of Danish Holstein dairy cattle for variant detection and insight into potential loss-of-function variants in protein coding genes
Source: BMC Genomics. 2015 Dec 9;16:1043. doi: 10.1186/s12864-015-2249-y (PMC4673847; doi:10.1186/s12864-015-2249-y)

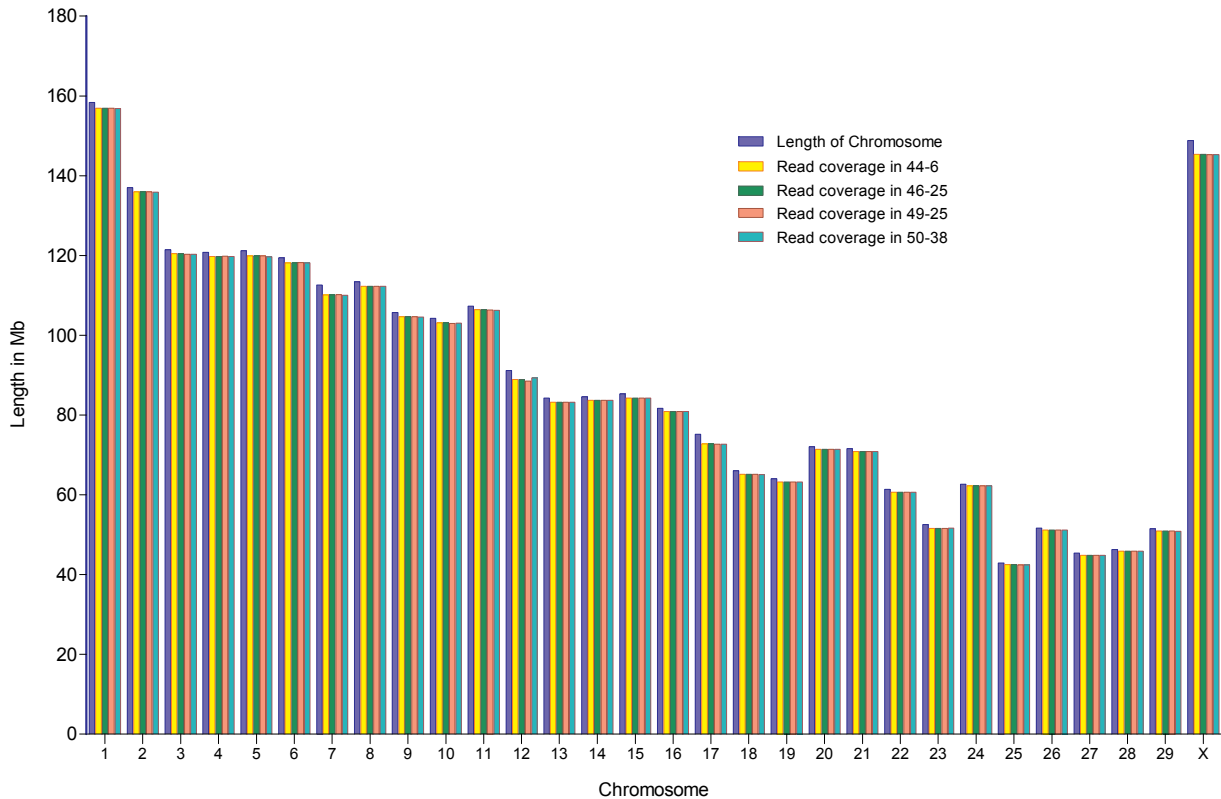

Supplement: Additional file 1: — Per chromosome coverage by sequencing reads. The horizontal axis shows 29 autosomes and X chromosome of the reference genome while the left vertical axis indicates scale of the chromosome length in Mbp. (PDF 23 kb) [file 12864_2015_2249_MOESM1_ESM.pdf]

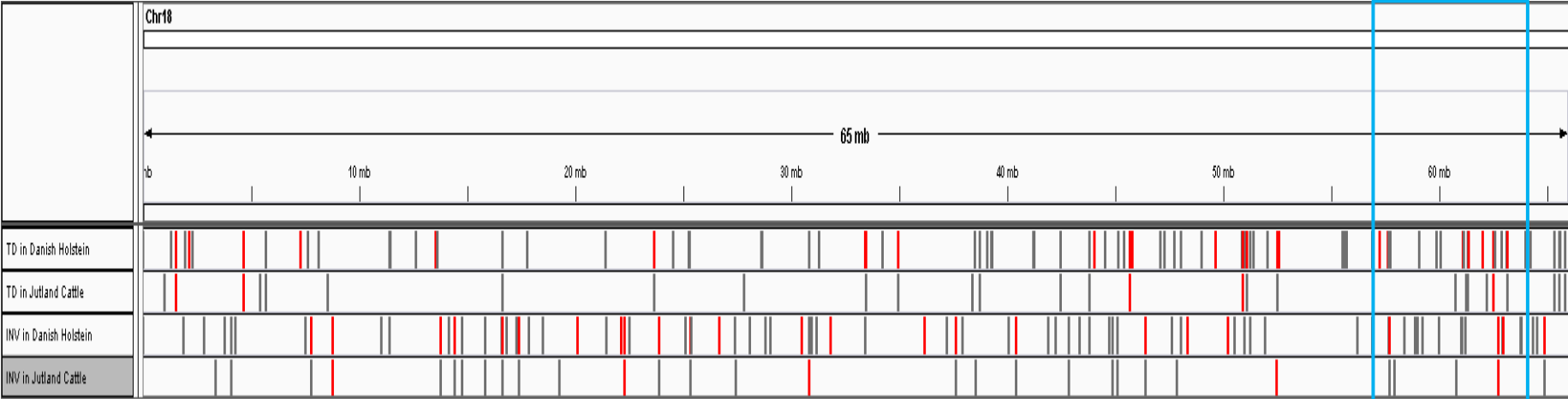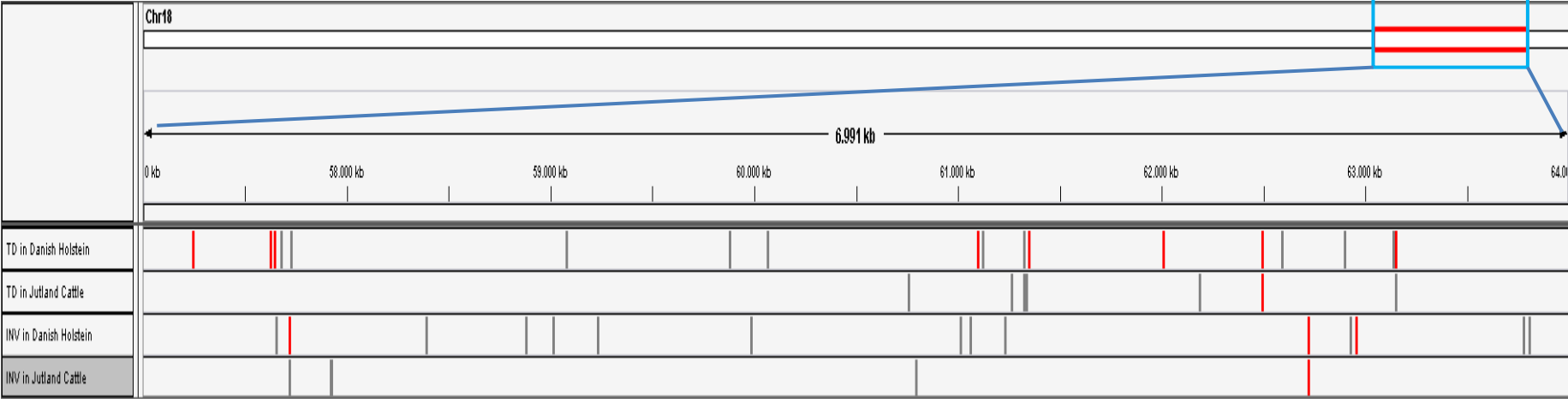

Supplement: Additional file 4: — Structural variants in BTA 18 (TD tandem duplications and INV inversions). Vertical grey bars indicate heterozygous variants while the red bars indicate homozygous variants. The blue mark highlighted region represents BTA18:57–64 Mb. (PDF 8 kb) [file 12864_2015_2249_MOESM4_ESM.pdf]
